# Direct Redox Sensing of Caffeine Utilizing Zinc-Doped Tin Oxide Nanoparticles as an Electrocatalyst

**DOI:** 10.34133/bmef.0099

**Published:** 2025-02-19

**Authors:** Gaurav Bhanjana, Ravinder Lamba, Manjit Singh Jadon, Neeraj Dilbaghi, Sandeep Kumar

**Affiliations:** ^1^ Department of Bio and Nano Technology, Guru Jambheshwar University of Science and Technology, Hisar, Haryana 125001, India.; ^2^ Department of Physics, Guru Jambheshwar University of Science and Technology, Hisar, Haryana 125001, India.; ^3^ Department of Physics, Punjab Engineering College (Deemed to be University), Chandigarh 160012, India.

## Abstract

**Objective:** In addition to its positive benefits, caffeine also has harmful consequences. Therefore, it is essential to ascertain its content in various substances. **Impact Statement:** The present study emphasizes a novel way of quantification of caffeine in real as well as laboratory samples based on a nanomaterial-assisted electrochemical technique. **Introduction:** Electrochemical sensing is a prominent analytical technique because of its efficiency, speed, and simple preparation and observations. Due to its low chemical potential, SnO_2_ (tin oxide) demonstrates rapid redox reactions when used as an electrode. The presence of shielded 4f levels contributes to its distinctive optical, catalytic, and electrochemical capabilities. **Methods:** An efficient coprecipitation approach, which is simple and rapid and operates at low temperatures, is utilized to produce zinc-doped tin oxide nanoparticles (Zn–SnO_2_ nanoparticles). Zinc doping is used to modify the optoelectronic characteristics of tin oxide nanoparticles, rendering them very efficient as electrochemical sensors. **Results:** The crystal structure of samples was analyzed using x-ray diffraction, electronic transitions were calculated using ultraviolet–visible spectroscopy, and surface morphology was analyzed using field emission scanning electron microscopy. The x-ray diffraction investigation revealed that the produced Zn-doped SnO_2_ nanoparticles exhibit tetragonal phases, and the average size of their crystallites reduces upon doping Zn with SnO_2_. The bandgap energy calculated using the Tauc plot was found to be 3.77 eV. **Conclusion:** The fabricated caffeine sensor exhibits a sensitivity of 0.605 μA μM ^−1^ cm^−2^, and its limit of detection was found to be 3 μM.

## Introduction

Nanomaterials with exotic properties compared to their bulk counterparts play a vital role in emerging sensor technology. Several sensor probes have been fabricated with the aid of biomedical engineering for quantification of various biological and chemical species [1–3]. Tin oxide, an important n-type semiconductor, has attracted considerable interest because of its unique properties and broad range of uses in different fields such as optoelectronic devices, energy storage, catalysis, solar cells, biomedical imaging, and electrochemical applications [4–6]. Electrochemical sensing is becoming increasingly popular in many areas, such as environmental monitoring and healthcare applications. Electrochemical sensors provide precise and very sensitive identification of analytes in complex samples. Tin oxide (SnO_2_) nanoparticles possess exceptional electrical conductivity, superior thermal stability, and remarkable optical characteristics, rendering them an optimal selection for electronic devices and sensors [7]. Due to its low chemical potential, SnO_2_ demonstrates rapid redox reactions when used as an electrode. The presence of shielded 4f levels contributes to its distinctive optical, catalytic, and electrochemical capabilities [8]. The existence of oxygen vacancies in tin oxide plays a vital role in its electrochemical use. However, its highly resistive nature diminishes its overall performance. Zinc (Zn) is used as a dopant to improve the electrochemical characteristics and electrical conductivity of SnO_2_ [9]. Within the human population, the ingestion of caffeine has a lengthy history due to its appealing flavor and ability to enhance both mental and physical activity. Caffeine is an inherent alkaloid present in various plant species, like coffee beans, cocoa beans, tea leaves, and guarana berries. Furthermore, caffeine is included in both energy beverages and medicinal medications. Due to its beneficial impact on physical endurance, concentration enhancement, reduction of oxidative stress, and alleviation of weariness and headache, caffeine is used in substantial amounts [10]. Nevertheless, the overconsumption of caffeine results in detrimental consequences such as anxiety, elevated blood pressure, and cardiovascular ailments. Due to the extensive use of caffeine-containing items, caffeine is introduced into municipal wastewater through urine. Approximately 20 metabolites are identified as products of caffeine biotransformation in the liver [11,12]. It is crucial to comprehend the potential hazards associated with the consumption of caffeine. Aside from the numerous medical conditions that humans can encounter such as high blood pressure, a fast heart rate, insomnia, and difficulty falling asleep, people who try cutting back on or giving up coffee also endure withdrawal symptoms, such as headaches and mood swings. Having reliable methods to identify caffeine in a variety of goods is essential to address these issues and guarantee people’s safety [13]. Because of the great sensitivity and selectivity for processing real-time data, electrochemical sensing techniques have shown substantial promise in the past several years in a variety of applications involving the detection of caffeine. The electrochemical sensing process involves use of the interaction between a working electrode and a material (analyte) to generate an electrical signal that is then used for analysis. The presence and quantity of the substance can be determined by measuring this signal. Several advantages come from using electrochemical sensors to detect caffeine. Electrochemical sensor devices are quick to respond, lightweight, and portable. These sensors are adaptable and have a wide range of applications, including monitoring caffeine content in drinks, dietary supplements, and medications [14–17].

## Results

**Fig. 1.**
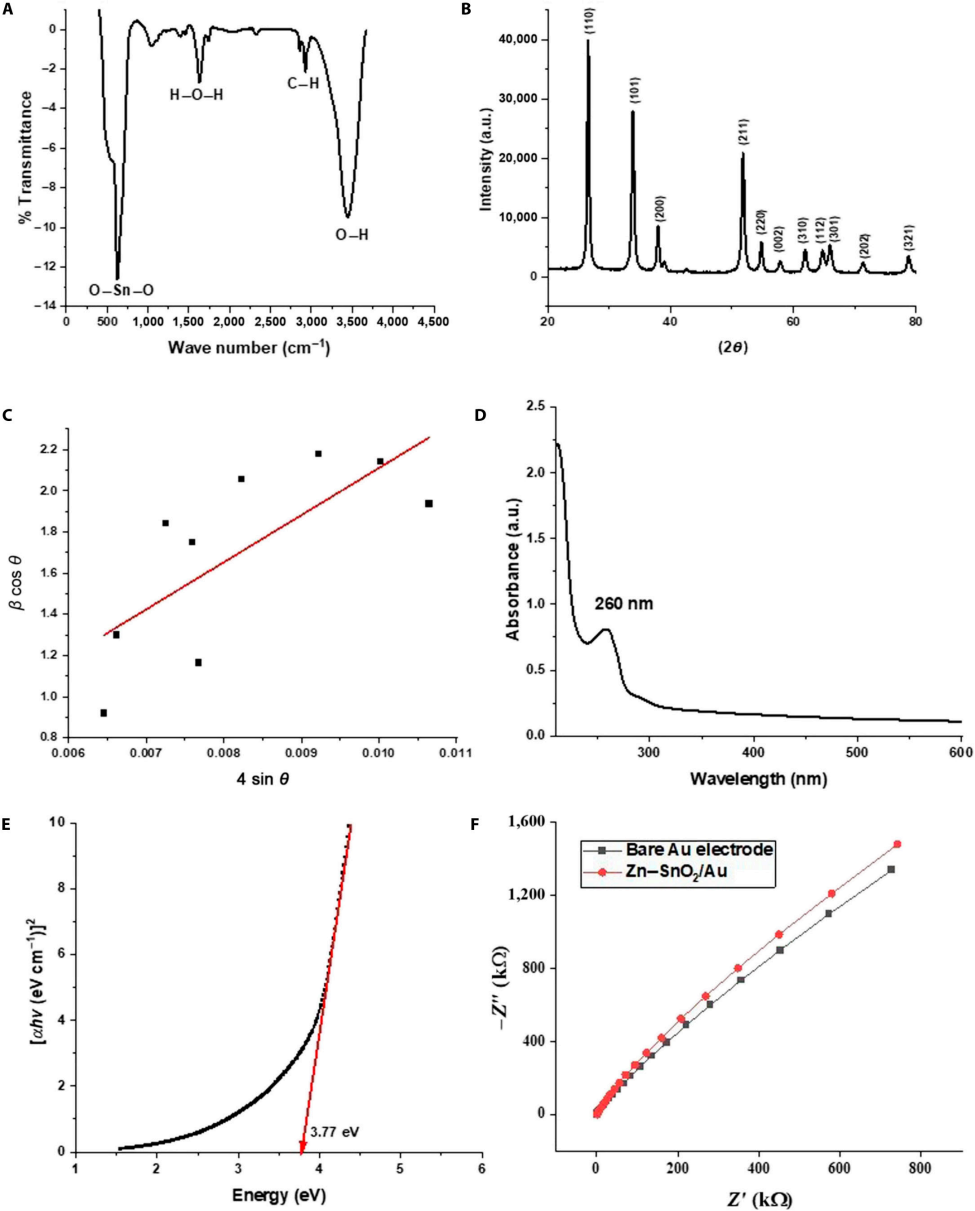
(A) Fourier transform infrared (FTIR) spectroscopy, (B) x-ray diffraction (XRD), (C) Williamson–Hall (W-H) plot, (D) ultraviolet (UV) absorbance spectra, (E) Tauc plot, and (F) electrochemical impedance spectroscopy (EIS) Nyquist plots of modified Zn–SnO_2_/Au and bare Au electrodes.

The various characterizations of Zn-doped SnO_2_ nanoparticles are shown in Fig. 1. For example, Fig. 1A shows the results of Fourier transform infrared (FTIR) spectroscopy; Fig. 1B and C show x-ray studies and a Williamson–Hall (W-H) plot, respectively; Fig. 1D and E show ultraviolet (UV) absorption spectroscopy and a Tauc plot, respectively, to determine the bandgap of nanoparticles; and Fig. 1F shows the electrochemical impedance spectroscopy study of the fabricated electrode and bare electrode.

**Fig. 2.**
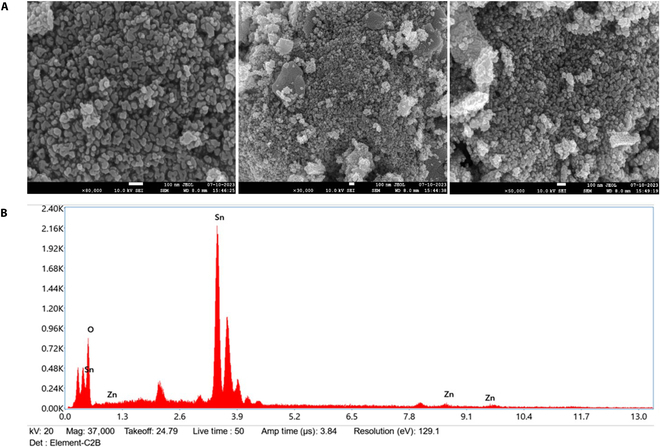
(A) Field emission scanning electron microscopy (FESEM) images and (B) energy-dispersive x-ray analysis (EDAX) spectra of Zn-doped SnO_2_ nanoparticles.

Figure 2A presents the photographs taken with a field emission scanning electron microscope to view the shape and size of the nanoparticles, while Fig. 2B demonstrates energy-dispersive x-ray analysis, a method that employs x-rays to determine the elemental composition of materials.

**Fig. 3.**
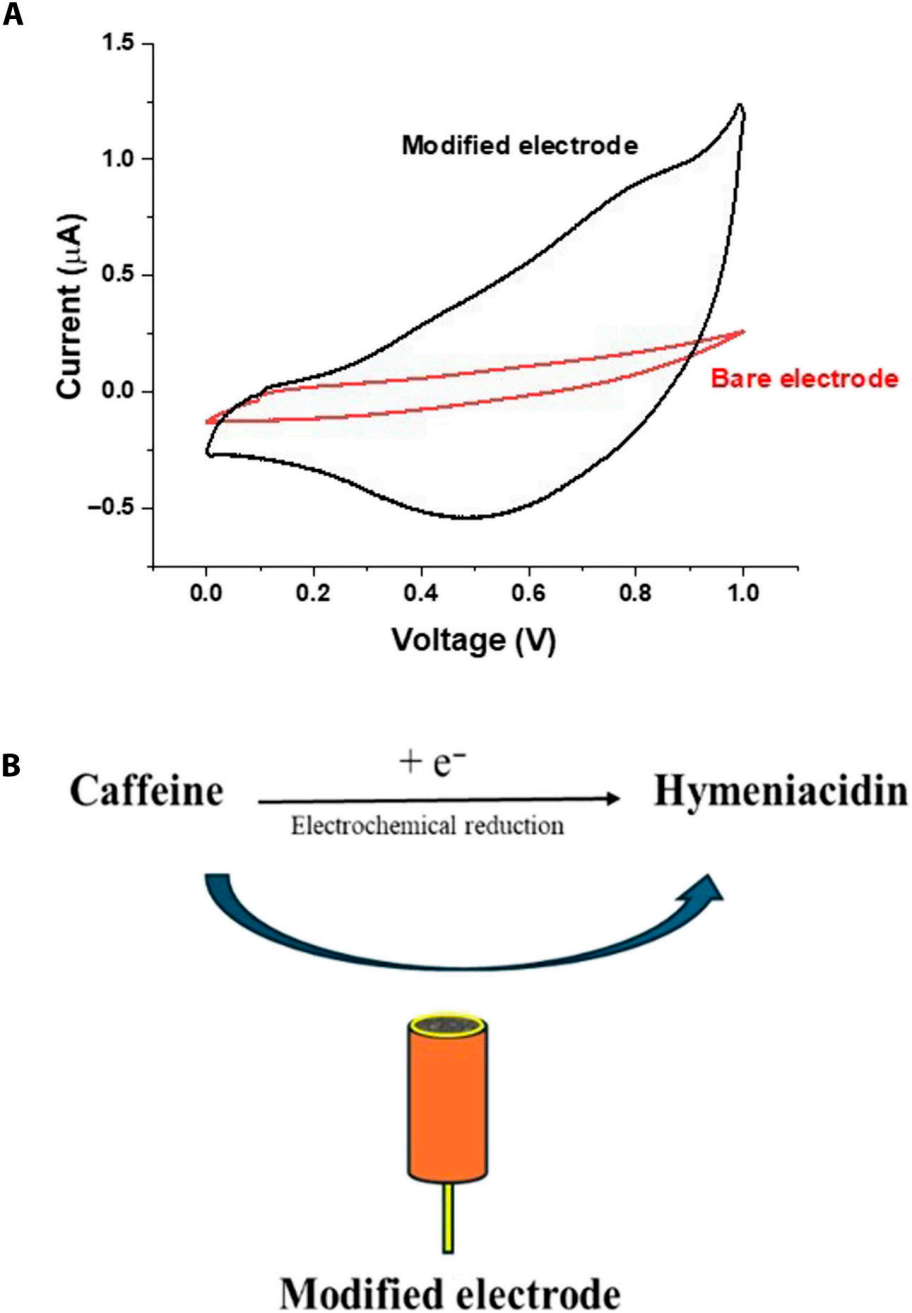
(A) *C*–*V* of the modified electrode versus that of the bare electrode in caffeine solution; (B) proposed electrochemical reduction of caffeine.

Figure 3A presents the cyclic voltammetric (CV) response of the modified or unmodified electrode in the presence of the analyte, and Fig. 3B presents the proposed mechanism of reduction of caffeine in the presence of the modified electrode.

**Fig. 4.**
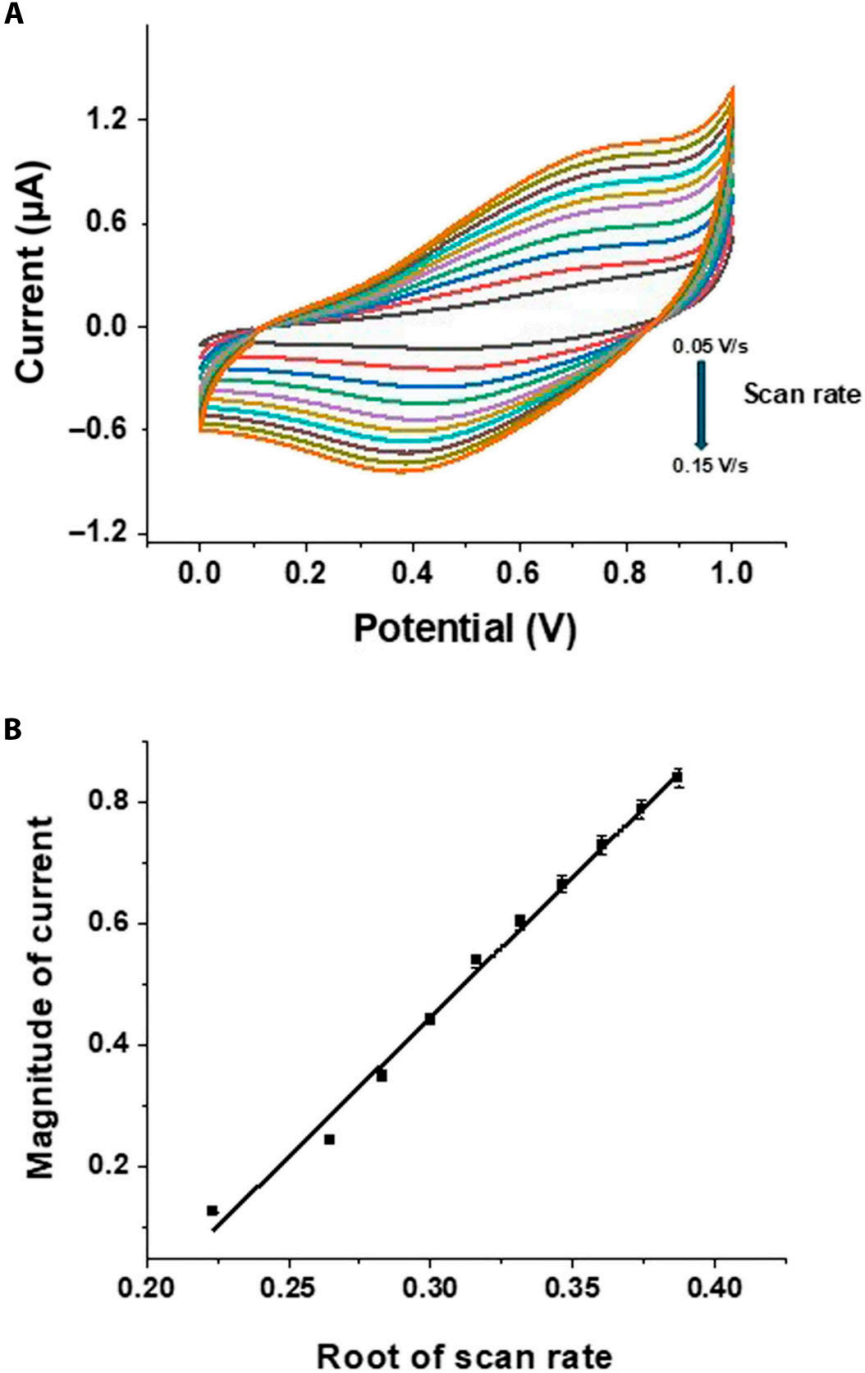
(A) Scan rate’s influence on the *C*–*V* response of the modified electrode. (B) Scan rate’s effect on the peak current on the *C*–*V* response of the electrode (*n* = 3).

Figure 4 presents the effect of varying the scan rate of the potential on the CV response of the modified electrode in the presence of the analyte.

**Fig. 5.**
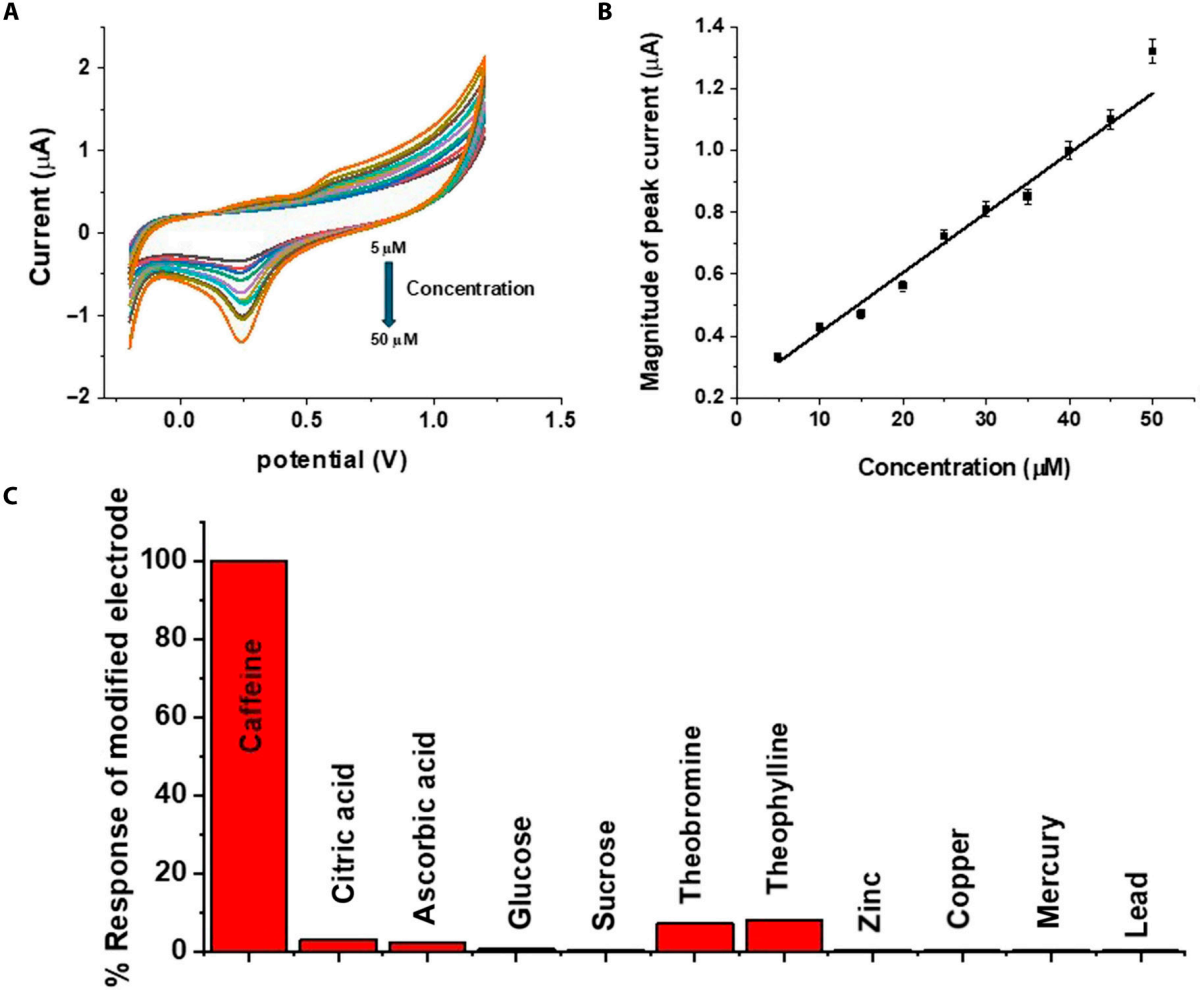
(A) Concentration’s effect on the *C*–*V* response of the modified electrode. (B) Concentration’s effect on the peak current on the *C*–*V* response of the electrode (*n* = 3). (C) Interference results for various possible interfering species (the concentration of the interfering species is 0.01 mM).

**Fig. 6.**
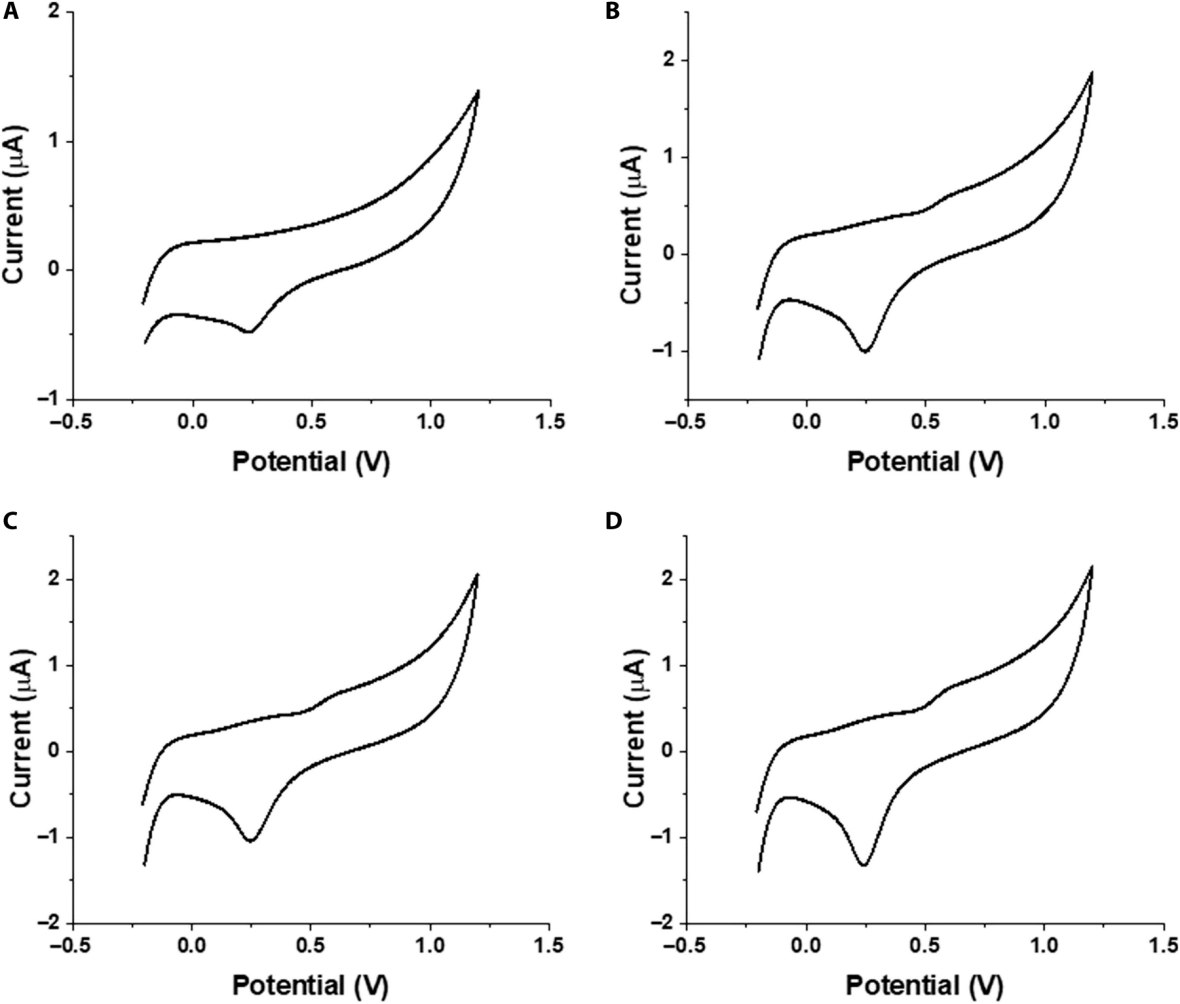
*C*–*V* response of spiked samples with different concentrations of caffeine. (A) Spiked canal water; (B) spiked groundwater; (C) spiked reverse osmosis (RO) water; (D) spiked drinking water supply.

Figure 5A and B show how the analyte concentration affects the peak current value, whereas interference studies are shown in Fig. 5C. Figure 6 shows the built sensor’s CV response in various caffeine-spiked samples.

## Discussion

### FTIR spectroscopy

Quantitative information on Zn-doped tin oxide nanoparticles was studied with FTIR spectroscopy. The data obtained revealed the presence of Sn–O, C–H, and O–H vibrational modes, indicating the presence of hydroxyl groups on the surface of the tin oxide nanoparticles. The peak near 3,452 cm^−1^ belongs to the O–H bond, while the peaks at 1,634 cm^−1^ correspond to H–O–H bond bending, and those at 2,856 and 2,925 cm^−1^ correspond to C–H bond bending [18]. The prominent peak at 624 cm^−1^ corresponds to O–Sn–O vibration. The investigation revealed no impurities in the tin oxide nanoparticles, and the formation of the Sn–O bond was indicated by a prominent peak at 620 cm^−1^ [19].

### X-ray diffraction

One of the most important characterization techniques for determining the phase, average crystallite size, and crystalline structure of the produced nanoparticles is x-ray diffraction (XRD). The Zn-doped SnO_2_ XRD spectra are shown in Fig. 1B. The XRD lines exhibit a clear indication of Zn successfully substituting into the host structure due to the changing of peak locations and enhancement in peak strength with doping. The radius of Zn^2+^ is 0.74 Å, while that of Sn^4+^ is 0.69 Å. The SnO_2_ nanoparticles crystallization planes (110), (101), (200), (211), (220), (002), (310), (112), (301), (202), and (321) correspond to the diffraction peaks found at 2*θ* = 26.54°, 33.84°, 37.90°, 51.80°, 54.80°, 57.86°, 61.84°, 64.68°, 65.98°, 71.24°, and 78.64°, respectively, which validates the formation of the cassiterite structure of Zn-doped SnO_2_ compounds [20,21]. The cassiterite structure of Zn-doped SnO_2_ compounds is related to the tetragonal structure and 136: *P*42/*mnm* space group of SnO_2_. Peak shifting could be explained by Zn^2+^ ions entering the SnO_2_ crystal planes and causing lattice distortion, which shows that Zn is clearly bonded to the SnO_2_ lattice [19]. The Debye–Scherrer equation (Eq. 1) was used to get the average crystallite size (*D*):

$$D=\frac{K\lambda }{\beta \text{cos}\theta }$$ (1)

where *K* (constant) = 0.9, *λ* (x-ray wavelength of Cu Kα) = 1.5418 Å, *θ* is the diffraction angle, and *β* is the full width at half maximum intensity. The average crystallite size (*D*) was calculated using the Debye–Scherrer equation for Zn-doped SnO_2_ nanoparticles and was found to be 17.38 nm (Table 1). Bragg’s law defines the lattice constant. The obtained lattice constants are *a* = *b* = 4.74 Å and *c* = 3.18 Å. The lattice constants increase slightly with doping, which can be caused by the dopant-related stress and strain effect.

**Table 1.** Calculated size and FWHM corresponding to different peaks

2*θ*	26.54	33.84	37.9	51.8	54.8	57.86	61.84	64.68	65.98
FWHM	0.38	0.46	0.401	0.484	0.468	0.697	0.55	0.68	0.63
Size (nm)	21.47	18.044	20.938	18.239	19.112	13.017	16.830	13.822	15.028

FWHM, full width at half maximum

To examine the distinct effects of crystallite sizes and lattice strain on peak broadening, the W-H method is utilized. The particle size is found to be 33.23 nm when calculated using the W-H plot; this clearly show the lattice strain effect on peak broadening.

### UV–visible spectroscopy

The Zn-doped SnO_2_ nanoparticles were characterized using UV–visible (UV–Vis) spectroscopy, and the results are presented in Fig. 1D. In order to do this, a small quantity of the Zn-doped tin oxide nanoparticles was introduced into sample vials containing double-distilled (DD) water and subjected to sonication for a duration of 20 min to guarantee proper dispersion of the solvent. The absorption maximum (*λ*_max_) of the nanoparticle occurs at a wavelength of 260 nm. The Tauc relation was employed to estimate the bandgap (*Eg*) of the samples by analyzing the plot of (*αhυ*)^2^ vs. *hυ*, as illustrated in Fig. 1E. The point at which the linear part of the curve intersects the *hυ* axis provides the value of the sample’s *Eg*. The algorithm for estimating the bandgap using UV–Vis measurements is provided below (Eq. 2):

$$\left(\alpha h\upsilon ){}^{2}=(h\upsilon -Eg\right)$$ (2)

The energy difference between the valence band and the conduction band of Zn-doped SnO_2_ is 3.77 eV. The addition of Zn reduces the size of the crystallite, leading to an increase in the bandgap. By introducing Zn dopant ions into the SnO_2_ lattice, additional degenerate energy levels are introduced, and the Fermi level is elevated to the conduction band edge. This leads to a considerable enhancement in the total bandgap [22,23].

### Field emission scanning electron microscopy

Field emission scanning electron microscopy (FESEM) was employed to analyze the dimensions and morphology of the zinc-doped tin oxide nanoparticles that were produced. The obtained data exhibited the spherical morphology, uniform size distribution, and little aggregation of the synthesized zinc-doped tin oxide nanoparticles as shown in Fig. 2A. The crystallite size ascertained through XRD analysis aligns with the estimated average particle size. The diameter of zinc-doped tin oxide nanoparticles was confirmed to be in the range of 40 to 60 nm using scanning electron microscopy analysis.

### Voltammetric study of the fabricated sensor

The findings of an impedance measurement on both bare gold electrodes and fabricated Zn–SnO_2_/Au electrodes using a potentiostat are shown in Fig. 1F. Compared to the bare gold electrode, the modified electrode has a greater impedance. Better sensitivity, selectivity, noise reduction, and additional information about the electrochemical process are all facilitated by this greater impedance [24]. CV was employed to examine the voltammetric response of caffeine; a solution of 5 μM caffeine including a pinch amount of KCl used as a supporting electrolyte was subjected to analysis. The findings demonstrated that the utilization of modified electrodes yielded a more robust reaction to caffeine, whereas the bare electrode exhibited no response, as depicted in Fig. 3A [25].

Caffeine can be easily electrochemically oxidized; in fact, despite the abundance of studies on the topic in the literature, electrochemical reduction of caffeine is hardly ever documented. Thus, following a thorough examination using CV to determine the ideal experimental parameters for this electrochemical reduction, we performed cathodic reduction of caffeine. The suggested electrochemical reduction of caffeine on the modified electrode is shown in Fig. 3B [26]. Caffeine in aqueous medium gains an electron and reduces to hymeniacidin, with a notable reduction peak observed at almost −0.3 V.

### Influence of scan rate

Figure 4A displays the CV measurements of a 5 μM caffeine solution using Zn–SnO_2_/gold as an electrode with a varying scan rate. The CV measurements were obtained at different scan rates. When the scan rate was increased from 0.05 to 0.15 V/s, the cathodic peak current exhibited a linear increase, and the peak potential shifted toward negative values. This observation confirms the irreversibility of caffeine reduction. The current at the reduction peak exhibited a direct proportionality to the square root of the scan rate. The reduction process is controlled by diffusion as indicated by the CV response, which varies linearly with the scan rate as shown in Fig. 4B [27].

### Influence of concentration

The CV response was used to record the dependence of the reduction peak current of the electrode at various caffeine concentrations. Figure 5A displays the CV measurements of a varying concentration of caffeine from 5 to 50 μM using Zn–SnO_2_/Nafion/gold as an electrode; there is a linear increase in current with concentration as shown in Fig. 5B. The prepared caffeine sensor demonstrates a high sensitivity of 0.605 μA μM ^−1^ cm^−2^ and the limit of detection is found to be 3 μM, which are determined by dividing the slope of the standard curve with the surface area of the modified electrode and dividing the standard deviation by the slope of the standard curve and multiplying by 3, respectively [28,29].

### Interference studies

Potential interference from substances similar to caffeine (citric acid, ascorbic acid, glucose, sucrose, theobromine, and theophylline) was also appraised and is given in Fig. 5C. In addition to these, the other possible interfering agents present in real water samples were also considered during the interference studies and are presented in Fig. 5C. It is quite clear from the results that the modified sensor showed almost negligible interference from potential interfering species.

### Caffeine analysis in real samples

The constructed sensor was also used to analyze actual spiked samples. Figure 6 displays the CV responses to environmental (actual) samples that were spiked with a known quantity of caffeine and obtained from a variety of sources (such as tap water, groundwater, and canal water). Real samples were analyzed as such after spiking without any pre-treatment. Detailed analysis of potential interferences from other agents present in real-world samples was performed and is provided in Fig. 5C. The constructed electrode waws found to be unresponsive to a variety of species found in real water samples. Table 2 presents the actual sample sources, the spiking amounts of caffeine, and the amounts calculated using the fabricated sensor.

**Table 2.** Quantitative analysis of artificially contaminated real samples with caffeine

Sr. no.	Sample	Amount of caffeine detected by modified electrode (μM)	Spiking amount of caffeine (μM)
1	Spiked canal water (A)	13.65	14
2	Spiked ground water (B)	50.89	52
3	Spiked RO water (C)	53.75	54
4	Spiked drinking water supply (D)	68.59	70

### Comparative study with previously reported literature

The modified sensor used in the present technique was compared for its performance with previously reported sensors. The comparison data are provided in Table 3. It is quite evident that the present technique exhibits excellent figures of merit compared with previously reported (worldwide) research studies. Hence, the present technique can be efficiently employed for real and lab sample electrochemical testing of caffeine.

**Table 3.** Comparative analysis of the present sensor with previously reported sensors

Sr. no.	Working electrode used	LOD (μM)	Linear range (μM)	Method of detection	Reference
1	Graphite raw cork and graphite regranulated cork-modified electrode	2.9 and 6.05	2.5–1,000	DPV	[30]
2	Cassava starch–Fe_3_O_4_ nanoparticle-modified glassy carbon electrode	23	500–900	DPV	[31]
3	Glassy carbon electrode modified with an electropolymerized film of para amino benzene sulfonic acid	11.95	10–100	SWV	[32]
4	Acid-activated carbon-nanofiber-modified glassy carbon electrode	17.40	25–450	SWV	[33]
5	Multiwall carbon nanotube immobilized glassy carbon electrode	3.54	10–110	DPV	[34]
6	Thiolated polyaniline, multiwalled carbon nanotube and gold–platinum core–shell nanoparticle-modified glassy carbon electrode	5.0	5–520	DPV	[35]
7	Carbon black, graphene oxide, copper nanoparticles and poly(3,4-ethylenedioxythiophene)–poly(styrenesulfonate)-modified glassy carbon electrode	3.4	11–64	CV/SWV	[36]
8	Zinc doped tin oxide-nanoparticle-coated gold electrode	3	5–50	CV	Present work

LOD, limit of detection; DPV, differential pulse voltammetry; SWV, square wave voltammetry; CV, cyclic voltammetry

## Conclusion

In conclusion, the coprecipitation method for the synthesis of zinc-doped tin oxide (Zn–SnO_2_) nanoparticles was utilized. XRD spectrometry confirmed the tetragonal phases of the Zn-doped SnO_2_ nanoparticles with a crystallite size of 33.23 nm. In UV–Vis spectroscopy, nanoparticles demonstrated a strong absorption at 260 nm, and a bandgap of 3.77 eV was calculated by using the Tauc plot. FESEM analysis provided valuable insights about the compositional and structural information of the synthesized nanoparticles. The size was observed to be 40 to 60 nm from FESEM images, which was also similar to that of XRD data. The prepared caffeine sensor showed a linear range response with an increase in the concentration of caffeine with a sensitivity of 0.605 μA μM^−1^ cm^−2^ and a detection limit of 3 μM in a linear range of 5 to 50 μM. Furthermore, the fabricated caffeine sensor exhibits substantial potential in real sample analysis free from influence of other compounds. Overall, this work advances the field of sensor technology and emphasizes the need to explore novel materials for various analytical applications. The reproducibility of the fabricated sensor was calculated using the relative standard deviation value, which was found to be 4%.

## Methods

Zinc-doped tin oxide nanoparticles were synthesized using SnCl_2_·2H_2_O and ZnSO_4_·7H_2_O as the respective sources of tin and zinc, which were procured from Sigma-Aldrich. All other chemicals used during the study were also purchased from Sigma-Aldrich. Water that had been twice deionized and 99.99% pure ethanol were the solvents utilized in the procedure. All chemical reactions were carried out in a round-bottom flask.

### Synthesis of Zn-doped SnO_2_ nanoparticles

Using the suitable precursors and the coprecipitation method, zinc-doped tin oxide (Zn–SnO_2_) nanoparticles were synthesized. To get started with doping, 0.1 M SnCl_2_·2H_2_O was dissolved in 50 ml of DD water and then ZnSO_4_·7H_2_O at a weight percentage of 0.02% was added to the mixture. The mixture was stirred at room temperature using a magnetic stirrer for 10 min. Thereafter, 0.2 M potassium hydroxide (KOH) solution was gradually added into the above prepared solution until it reached a pH of 10. The mixture was further stirred for 2 h at room temperature until precipitates of Zn-nanoparticle-doped SnO_2_ were formed. The formed precipitates were subjected to many cycles of centrifugation and washing with DD water and 99.99% pure ethanol. The obtained nanoparticles underwent a drying procedure at 60 °C in an oven for a period of 4 h. Finally, the nanoparticles were subjected to annealing in a muffle furnace at a temperature of 600 °C for 1 h, using a silica crucible.

### Characterization

XRD analysis was carried out using a Benchtop MiniFlex II model (Rigaku, Japan) at a wavelength of 0.154 nm and a scanning range from 10° to 80° (2*θ*). The UV–Vis spectra of nanoparticles were recorded with a UV–Vis–NIR spectrophotometer (Varian Cary 5000) in the range of 200 to 600 nm. Using KBr powder as the optical system, the FTIR spectra were acquired using a PerkinElmer Spectrum model. At a resolution of 0.5 cm^−1^, the spectra were obtained in the region of 4,000 to 400 cm^−1^. The FESEM images were acquired with a JEOL 7610F Plus model.

### Fabrication of the electrode and electrochemical studies

The synthesized zinc-doped tin oxide nanoparticles were coated on a gold electrode with the help of the binding agent Nafion. The fabricated electrode was evaluated for its electrochemical response toward caffeine using the conventional 3-electrode system of Metrohm (Autolab). The coated electrode was evaluated as the working electrode with respect to a platinum wire as the counter electrode and the reference Ag/AgCl electrode. Electrochemical impedance spectroscopy was performed to compare the impedance profile of the coated electrode with that of a bare Au electrode. All electrochemical experiments were carried out in the presence of KCl (aqueous solution) as the supporting electrolyte and at neutral pH. Various affecting parameters like scan rate, caffeine concentration, interference, and real sample analysis were also optimized during the present study.

### Statistical parameters

All figures were drawn with the help of Origin 9.1. The mean and standard deviation (*N* = 3) are presented in results with their respective data. The reproducibility of the fabricated sensor was calculated using the relative standard deviation value.

## Data Availability

The experimental data will be made available on request from the corresponding authors.
